# The difference of T cell phenotypes in end stage renal disease patients under different dialysis modality

**DOI:** 10.1186/s12882-019-1475-y

**Published:** 2019-08-05

**Authors:** Jiao Xiaoyan, Chen Rongyi, Cao Xuesen, Zou Jianzhou, Ji Jun, Ding Xiaoqiang, Yu Xiaofang

**Affiliations:** 1 Department of Nephrology, Zhongshan Hospital, Fudan University, NO180, Feng’lin Road, Shanghai, 200032 China; 2 Shanghai Medical Center for Kidney, Shanghai, China; 3 Shanghai Key Laboratory of Kidney and Blood Purification, Shanghai, China; 4 Shanghai Institute of Kidney and Dialysis, Shanghai, China

**Keywords:** End-stage renal disease, Peritoneal dialysis, Hemodialysis, T cell phenotypes

## Abstract

**Background:**

Impaired T cell immune function exists in end-stage renal disease (ESRD) patients. Dialysis treatment may lead to changes in T cell subsets. In the present study, we aimed to identify alterations of T cell phenotypes in ESRD patients, especially in those receiving peritoneal dialysis (PD), and analyze the potential associated factors.

**Methods:**

In the present study, 110 PD patients and 110 age/gender-matched hemodialysis (HD) patients who met the inclusion criteria were studied. Pre-dialysis blood samples were obtained and analyzed by flow cytometry to detect the expression of CD45RO and CCR7. Univariate and multivariate regression analyses were used to determine the factors associated with the alteration of T cell phenotypes.

**Results:**

In all dialysis patients, age was associated with the frequencies of both CD4+ and CD8+ naïve T cells, effector memory (EM) T cells and effector memory RA (EMRA) T cells but not central memory (CM) T cells. Dialysis modality was also associated with T cell subsets. Compared with HD patients, PD patients showed an increase in both CD4+ and CD8+ CM T cells and a reduction in both CD4+ and CD8+ EM and EMRA T cells. However, the number of CD4+ naïve T cells was lower and the number of CD8+ naïve T cells was higher in PD patients than those in HD patients. In PD patients, further multivariate analysis revealed that the frequency of CD4+ naïve T cells was positively associated with nPCR, while the frequency of CD8+ naïve T cells was negatively associated with age.

**Conclusion:**

In dialysis patients, the dialysis modality and age influence T cell subsets. There is a progression from naïve to effector T cells in HD patients compared with PD patients. In PD patients, different factors may influence the frequencies of CD4+ and CD8+ naïve T cells.

## Background

End-stage renal disease (ESRD) is associated with immune dysfunction, characterized as systemic inflammation and immune deficiency [1, 2]. The immunological abnormalities increased the risk of cardiovascular disease (CVD) and susceptibility to infection and cancer, accounting for high morbidity and mortality in patients with ESRD [3, 4].

T cells, the major mediator of adaptive immunity, can be classified according to the surface markers (CD45RO and CCR7) into four subsets: naïve, central memory (CM), effector memory (EM) and effector memory RA (EMRA) T cells [5]. Upon antigen stimulation, naïve T cells expand and differentiate into long-lived memory T lymphocytes and shorted-lived effector T lymphocytes. The long-lived CM T cells can rapidly expand in response to secondary challenge, whereas effector T cells exert specific functions [5, 6].

In ESRD patients, T-cell mediated immune dysfunction results from an inverted CD4+/CD8+ ratio, marked loss of naïve T cells and accumulation of differentiated T cells [7–9]. With the loss of renal function, the abundance of naïve T cells decreases [8, 10]. The reduction in the number of naïve T cells leads to an inadequate immune response to antigen [11, 12]. Hemodialysis (HD) and peritoneal dialysis (PD) have different effects on CD4+ T cell phenotypes and proliferation parameters [13]. However, the effect of dialysis modality, particularly the effect of PD on T cell subsets, has not been fully discussed.

Therefore, we hypothesized that T cell phenotypes could be influenced by different dialysis modalities. We also investigated the factors associated with the frequency of naïve T cells in PD patients.

## Methods

### Patients

In the present study, ESRD patients with stable dialysis for at least 3 months were recruited from January 1, 2016 to June 30, 2017. The patients with malignancy, acute and chronic infection (including the infection of common bacteria, hepatitis B virus, hepatitis C virus and human immunodeficiency virus), kidney transplantation, and use of immunosuppressive drugs within the past 3 months were excluded.

The 110 enrolled PD patients, aged between 18 and 85 years old, were treated on standard continuous ambulatory peritoneal dialysis (CAPD) with glucose-based PDF (Dianeal®; Baxter). For every PD patient, one age- and gender-matched HD patient was included. All HD patients were undergoing dialysis for at least 4 h thrice weekly. Hemodialysis was performed with standard dialysate by low-flux dialyzers. All patients remained on the initial dialysis modality.

The study and protocol were reviewed and approved by the Medical Ethics Committee of Zhongshan Hospital, Fudan University.

### Preparation of cells

Fasting blood samples were obtained from a peripheral vein before dialysis. Subsequently, the whole blood samples were lysed by red blood cell lysis solution (10 mM KHCO3, 155 mM NH4Cl from Sangon Biotech, and 0.1 mM EDTA from Sigma-Aldrich, pH =7.2). After washing twice, the cells were resuspended with staining buffer (PBS containing 0.2% FBS from Invitrogen and 0.09% NaN3 from Sigma-Aldrich).

### Flow cytometry analysis

**Fig. 1:**
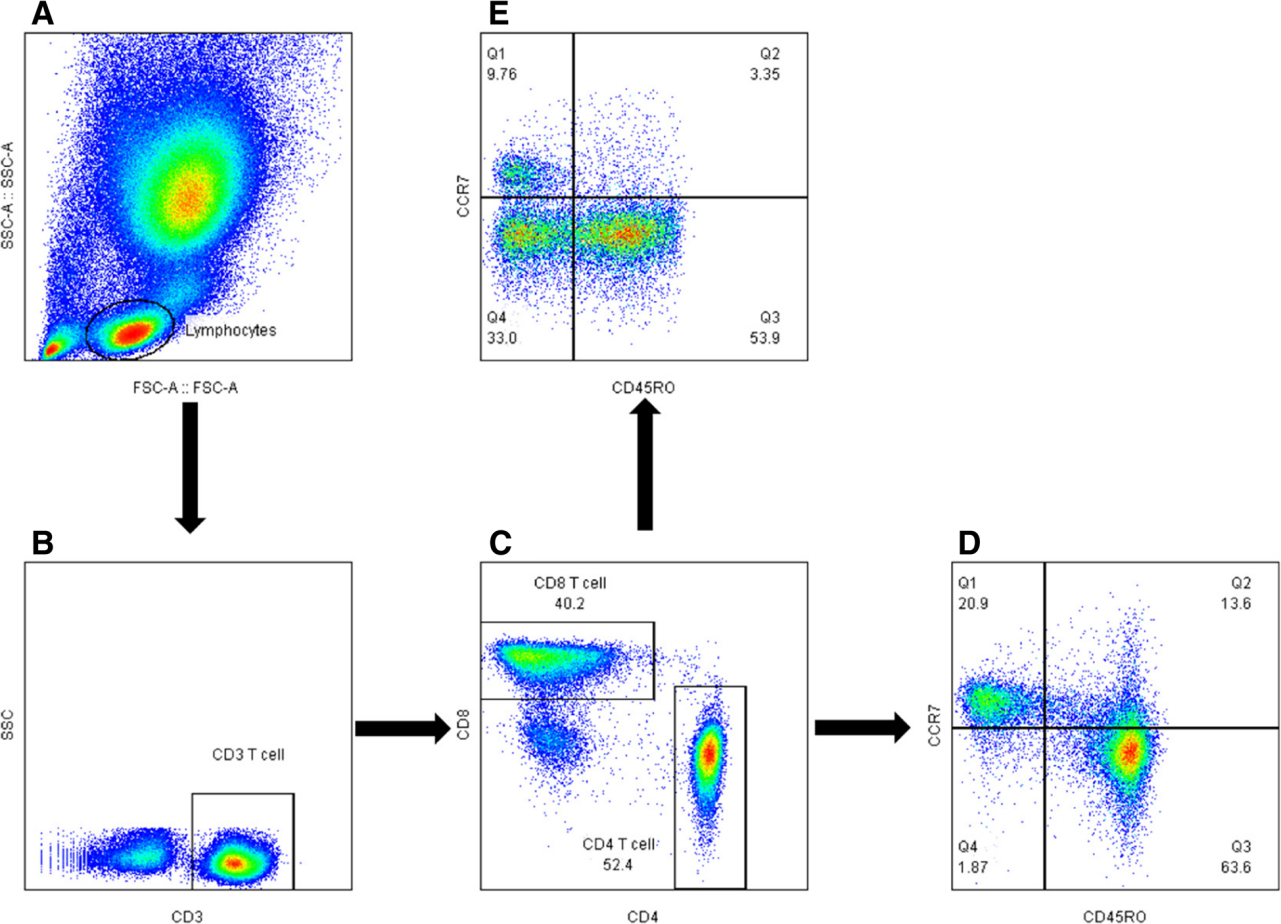
Flowchart of flow cytometry analysis to identify CD4 and CD8 T cells. **a** The flow cytometry picture of lysed peripheral blood including lymphocytes; **b** the flow cytometry picture of T cells gated from lymphocytes; **c** the flow cytometry picture of CD4 T cells and CD8 T cells gated from T cells; **d** the distribution of naïve T cells (CD45RO − CCR7+), central memory T cells (CM, CD45RO+ CCR7+), effector memory T cells (EM, CD45RO + CCR7−), EMRA (CD45RO − CCR7−) gated from CD4 T cells; **e** the distribution of naïve T cells (CD45RO − CCR7+), central memory T cells (CM, CD45RO+ CCR7+), effector memory T cells (EM, CD45RO + CCR7−), EMRA (CD45RO − CCR7−) gated from CD8 T cells

Peripheral blood cells in staining buffer were stained for 30 min at 4 °C and T lymphocytes was determined by staining of CD3-PE (Bio-Legend, San Diego, CA, USA) and CD4-APC or CD8a-PerCP/Cy5.5 (eBioscience, San Diego, CA, USA). FITC-labeled anti-CD45RO (Miltenyi Biotec, Bergisch Gladbach, Germany), and APC/Cy7-labeled anti-CCR7 (BioLegend) was used to identify the differentiation of T lymphocytes. The data were analyzed with a BD LSRFortessa™ flow cytometer (BD Bioscience, San Jose, CA, USA). Flow cytometry was used to assess markers of T cell subsets (Fig. 1). The surface markers CCR7 and CD45RO were used to classify the T cells into subsets: (1) naïve cells (CD45RO-, CCR7+); (2) central memory cells (CD45RO+, CCR7+); (3) effector memory cells (CD45RO+, CCR7-); and (4) effector memory RA cells (CD45RO-, CCR7-).

### Data analysis and statistics

The normally distributed variables were expressed as the means ± standard deviation. Skewed variables are shown as the median and interquartile range (IQR). Comparisons were performed by Student’s t test or the Mann–Whitney U test according to the normality of data. Pearson or Spearman’s correlation analysis was performed to explore the related factors for T cell subsets. Univariate and multivariate regression models were constructed to determine the associations between variables. All analyses were performed with SPSS (Chicago, IL, USA), version 23.0. The statistical significance level was set to 0.05 in 2-tailed testing.

## Results

### Characteristics of patients

Demographic and clinical characteristics are summarized in Table 1. Dialysis vintage of HD patients was significantly longer than that of PD patients (*p* < 0.001). The underlying kidney diseases of PD patients were chronic glomerulonephritis (24.8%), hypertensive nephropathy (17.3%) and diabetic kidney disease (15.8%), whereas the major cause in HD patients was glomerulonephritis (47.4%), followed by diabetic kidney disease (19.8%) and hypertensive nephropathy (9.9%).

**Table 1:** Baseline characteristics of peritoneal dialysis patients and hemodialysis patients

Characteristics	PD	HD	*p*
Number	110	110	
Median age (year)	57.5±14.4	56.0±14.7	0.263
Gender, n (%)			
Male	53 (48.2)	53 (48.2)	0.844
Female	58 (52.8)	58 (52.8)	
Dialysis vintage, months	14.5 (9, 31)	46 (28, 73)	< 0.001
CMV exposure (%)	92.8	91.5	0.88
Underlying kidney disease, n (%)			
Chronic glomerulonephritis	33 (24.8)	53 (47.7)	0.006
Hypertensive nephropathy	23 (17.3)	11 (9.9)	0.025
Diabetic kidney disease	21 (15.8)	22 (19.8)	0.865
Kidney stone disease	3 (2.2)	2 (1.8)	0.651
Polycystic kidney disease	5 (3.8)	4 (3.6)	0.734
Others	31 (23.3)	14 (12.6)	0.002
Unknown	17 (12.8)	5 (4.5)	0.007
Laboratory parameters			
WBC (× 10^9^/L)	6.4 (5.2, 7.7)	6.3 (5.1, 7.88)	0.134
Neutrophil (× 10^9^/L)	4.2 (3.3, 5.0)	4.1 (3.3, 5.7)	0.925
Lymphocyte (× 10^9^/L)	1.5 (1.0, 1.9)	1.2 (1.0, 1.7)	0.126
Monocyte (× 10^9^/L)	8.7 (7.2, 10.1)	8.0 (6.7, 9.4)	0.108
T cell (× 10^6^/L)	1075.5 (769.0, 1345.0)	800.0 (609.0, 992.5)	< 0.001
CD4 T cell (× 10^6^/L)	638.0 (486.0, 798.5)	452.0 (361.0, 586.0)	< 0.001
CD8 T cell (× 10^6^/L)	380.0 (220.3, 474.5)	310 (210.0, 423.5)	0.114
CD4/ CD8	1.96 (1.31, 2.58)	1.53 (1.31, 1.86)	0.038
Hemoglobin (g/L)	96.5 (80.0, 107.7)	111.0 (100.7, 121.0)	< 0.001
Albumin (g/L)	33 (31, 35)	39 (38, 41)	< 0.001
Pre-albumin (g/L)	0.33 (0.26, 0.38)	0.31 (0.24, 0.4)	0.402
Total cholesterol (mmol/L)	4.3 (3.7, 5.1)	4.1 (3.5, 4.7)	0.265
Triglyceride (mmol/dl)	1.3 (0.9, 2.2)	1.4 (1.0, 2.3)	0.285
ALT (U/L)	12.0 (9.0, 18.7)	6.0 (5.0, 9.2)	< 0.001
AST (U/L)	14.0 (11.0, 19.7)	19.0 (15.0, 27)	< 0.001
SCr (μmol/L)	904 (679, 1149)	991 (814, 1166)	0.026
BUN (mmol/L)	18.4 (15, 22.1)	27.2 (21.8, 31.1)	< 0.001
Calcium (mmol/L)	2.2 (2.1, 2.4)	2.3 (2.2, 2.5)	0.905
Phosphorus (mmol/L)	1.6 (1.3, 1.9)	2.3 (1.7, 2.6)	< 0.001
iPTH (pg/mL)	137.0 (78.9, 273.9)	263.5 (170.8, 384.2)	< 0.001
β2-MG (mg/L)	27.1 (21.5, 37.7)	39.1 (33.8, 43.5)	< 0.001
Iron (μmol/L)	10.0 (7.3, 13.0)	11.2 (8.9, 14.4)	< 0.001
Ferritin (μg/mL)	274.3 (135.4, 463.7)	301.8 (139.3, 494.0)	0.841
IL-6 (pg/ml)	6.3 (3.9, 11.0)	9.9 (3.7, 65.7)	0.014
TNF-α (pg/ml)	17.2 (13.7, 21.2)	30.0 (23.4, 61.4)	< 0.001
hsCRP (mg/L)	3.1 (1.6, 9.5)	3.9 (1.5, 8.0)	0.564

*WBC* White blood cells, *ALT* Alanine aminotransferase, *AST* Aspartate aminotransferase, *SCr* Serum creatinine, *BUN* Blood urine nitrogen, *β*_*2*_*-MG* β2-microglobin, *iPTH* Parathyroid hormone, *hsCRP* high-sensitive C-reactive protein

Considering the dialysis treatment adequacy, total Kt/V in PD patients was 1.82 (1.52, 2.31) while that in HD patients was 1.23 (1.1, 1.38). In PD patients, serum creatinine and blood urea nitrogen were lower than those in HD group. The serum levels of hemoglobin, albumin, β_2_-MG, AST, as well as phosphorus, iPTH and iron were significantly lower in PD patients than those observed in HD patients. However, serum TC, TG, calcium concentrations were similar between the two groups. PD patients showed a marked reduction of IL-6 and TNF-α in comparison to HD patients. hsCRP, ferritin level and the prevalence of cytomegalovirus infection (determined by detecting anti-CMV IgM and IgG antibodies in serum), did not differ significantly between PD and HD patients.

With respect of the total number of white blood cells, neutrophils, lymphocytes and monocytes, no difference was observed between PD and HD group. However, compared to HD group with a reduced number of CD4+ T cell and ratio of CD4 to CD8, the absolute T cell counts was higher in PD patients.

### Associations of age and dialysis modality with T cell subsets

To identify the factors that interact with T cell subsets in dialysis patients, a univariate general linear model was used to analyze the associations of age, gender, CVD, diabetes, dialysis modality, dialysis vintage and residual renal function (anuria) with the frequencies of T cells in the entire study population. As shown in Table 2, there was a significant interaction of age with the frequencies of naïve, EM and EMRA cells, but not CM T cells, among both CD4+ and CD8+ T cells. Dialysis modality was also significantly associated with the percentage of all the T cell subsets. However, T cell subsets were not affected by gender, CVD, diabetes, dialysis vintage or anuria.

**Table 2:** Factors associated with T cell subsets in ESRD patients

	Age	Gender	CVD	DM	Dialysis modality	Dialysis vintage	Anuria	R^2^
CD4 + T Cell								
Naïve CD4,%	0.027	0.732	0.584	0.382	0.002	0.987	0.884	0.060
CM CD4,%	0.858	0.091	0.505	0.674	< 0.001	0.199	0.141	0.311
EM CD4,%	0.001	0.099	0.935	0.155	< 0.001	0.153	0.286	0.157
EMRA CD4,%	0.023	0.124	0.951	0.993	< 0.001	0.848	0.451	0.092
CD8+ T Cell								
Naïve CD8,%	< 0.001	0.171	0.674	0.924	< 0.001	0.937	0.178	0.378
CM CD8,%	0.993	0.998	0.987	0.997	< 0.001	0.160	0.074	0.180
EM CD8,%	< 0.001	0.069	0.240	0.987	< 0.001	0.813	0.129	0.135
EMRA CD8,%	< 0.001	0.603	0.144	0.592	< 0.001	0.252	0.268	0.264

*CVD* Cardiovascular disease, *DM* Diabetes mellitus, *CM* Central memory, *EM* Effector memory, *EMRA* Effector memory RA

### T cell subsets in hemodialysis and peritoneal dialysis patients

Compared with HD patients, PD patients had a higher percentage of CD4+ CM T cells and a lower percentage of CD4+ naïve, EMRA and TEMRA T cells. In terms of CD8+ T cells, there was a similar distribution of higher CM and lower EMRA and TEMRA T cells in PD patients. However, the number of CD8+ naïve T cells in PD patients was higher than that in HD patients (Table 3).

**Table 3:** Comparison of T cell subsets between PD and HD patients

T Cell Subsets	PD(*n* = 110)	HD(n = 110)	*p*
CD4+ T Cell			
Naïve CD4, %	28.1(19.2, 41.3)	35.3(26.1, 46.8)	0.001
CM CD4, %	36.4(25.9, 51.3)	19.2(14.1, 26.2)	< 0.001
EM CD4, %	25.7(15.9, 33.6)	34.4(27.4, 45.2)	< 0.001
EMRA CD4, %	2.5(1.4, 4.9)	4.6(2.9, 8.8)	< 0.001
CD8+ T Cell			
Naïve CD8, %	40.2(19.6, 52.9)	21.4(13.0, 31.7)	< 0.001
CM CD8, %	6.3(2.8, 12.6)	2.4(1.5, 3.9)	< 0.001
EM CD8, %	31.9(17.9, 49.1)	50.7(37.7, 60.1)	< 0.001
EMRA CD8, %	14.2(8.4, 24.6)	20.5(13.9, 29.4)	< 0.001

*CM* Central memory, *EM* Effector memory, *EMRA* Effector memory RA

### Factors associated with naïve T cells in the PD group

Considering the different distributions of CD4+ and CD8+ naïve T cells in PD patients, we further explored factors that influence naïve T cells in PD patients.

In the univariate regression analysis, the nPCR was significantly positively associated with the frequency of CD4+ naïve T cells (Table 4). The frequency of CD8+ naïve T cells was negatively associated with age and positively associated with the serum albumin level, the nPCR, the serum phosphorus level, the serum calcium level, BUN and the SCr level (Table 5).

**Table 4:** Regression analysis for CD4+ naïve T cells in PD patients

	Univariate analysis		Multivariate analysis	
	*r*	*P*	*β* ^*a*^	*P*
Age (years)	0.032	0.743		
Gender (male)	0.045	0.642		
Albumin(g/L)	−0.048	0.618		
Pre-albumin (g/L)	0.047	0.624		
β_2_-MG, mg/L	0.079	0.460		
IL-6(pg/ml)	0.141	0.178		
TNF-α (pg/ml)	− 0.115	0.264		
hsCRP (mg/L)	0.147	0.132		
Calcium (mmol/L)	0.07	0.469		
Phosphorus (mmol/L)	0.16	0.093	0.191	0.073
Iron (μmol/L)	−0.02	0.835		
iPTH (pg/mL)	−0.021	0.083	−0.109	0.288
BUN (mmol/L)	0.136	0.153		
SCr (μmol/L)	0.151	0.114		
TC (mmol/L)	0.018	0.851		
TG (mmol/dl)	0.071	0.451		
nPCR (g/kg/day)	0.219	0.022	0.213	0.034
KtV	0.11	0.231		
Anuria	0.007	0.668		

*β2-MG* β2-microglobin, *hsCRP* high-sensitive C-reactive protein, *SCr* Serum creatinine, *BUN* Blood urine nitrogen, *iPTH* Parathyroid hormone, *BUN* Blood urea nitrogen, *SCr* Serum creatinine, *TC* Total cholesterol, *TG* Triglyceride, *nPCR* normalized protein catabolic rate

^a^In the backward stepwise multiple regression model, Adjusted *R*^2^ = 0.125.

**Table 5:** Regression analysis for CD8+ naïve T cells in PD patients

	Univariate analysis		Multivariate analysis	
	*r*	*P*	*β* ^*a*^	*P*
Age (years)	−0.47	< 0.001	−0.321	0.003
Gender (male)	0.027	0.778		
Albumin(g/L)	0.187	0.049	−0.062	0.544
Pre-albumin (g/L)	0.105	0.271		
β_2_-MG, mg/L	−0.053	0.619		
IL-6 (pg/ml)	0.036	0.73		
TNF-α (pg/ml)	−0.116	0.262		
hsCRP (mg/L)	0.014	0.889		
Calcium (mmol/L)	0.171	0.073	0.113	0.379
Phosphorus (mmol/L)	0.287	0.002	0.102	0.357
Iron (μmol/L)	0.034	0.731		
iPTH (pg/mL)	0.06	0.536		
BUN (mmol/L)	0.175	0.067	−0.041	0.728
SCr (μmol/L)	0.263	0.005	0.113	0.327
TC (mmol/L)	0.06	0.537		
TG (mmol/dl)	−0.054	0.582		
nPCR (g/kg/day)	0.222	0.02	0.174	0.111
Kt/V	−0.011	0.910		
Anuria	0.013	0.12		

*β2-MG* β2-microglobin, *hsCRP* high-sensitive C-reactive protein, *SCr* Serum creatinine, *BUN* Blood urine nitrogen, *iPTH* Parathyroid hormone, *TC* Total cholesterol, *TG* Triglyceride, *nPCR* normalized protein catabolic rate

^a^In the backward stepwise multiple regression model, Adjusted *R*^2^ = 0.277

Further multiple stepwise regression analyses indicated that the nPCR positively influenced the frequency of CD4+ naïve T cells (Table 4), while age was a negative independent factor influencing the frequency of CD8+ naïve T cells in PD patients (Table 5).

## Discussion

In the present study, we demonstrated that T cell subsets alteration were influenced by age and dialysis modality. T cell subsets in HD patients showed a more tendency to progress from naïve to effector cells than that in PD patients. Lower nPCR in PD patients contributed to the reduced frequency of CD4+ naïve T cells, while age was negatively associated with the frequency of CD8+ naïve T cells.

T cells alteration and dysfunction was associated with the progression of chronic kidney disease (CKD) [14]. The accumulation of uremic toxin and presence of inflammation milieu in ESRD patients accelerated the T cell premature aging, which was characterized as the reduction of naïve T cell but increased proportion of EMRA T cell [10, 15]. Considering the difference of inflammation milieu between HD and PD patients, their impact on the distribution of T cell phenotype has not been fully demonstrated. In the present study, we found that the dialysis modality was an important factor that influenced the proportion of T cells subsets. Consistent with other studies [8, 16], age was associated with alteration of T cell subsets in dialysis patients. We found the dialysis vintage and anuria were not related with the proportion of T cell subsets. Besides, as common complications or comorbidities in dialysis patients, DM and CVD also had no association with T cell subsets in our study.

The altered differentiation of T cells from naïve to effector T cells could occur with aging or cytomegalovirus infection [17, 18]. However, dialysis treatment may also prompt this alteration through decreasing the ability of T cell proliferation, exacerbating T cell apoptosis and reducing thymic output [7, 13, 19]. This alteration of T cell subsets may contribute to the attenuated response to antigen and increasing the risk of CVD in ESRD patients [11, 20, 21].

In the present study, a lower frequency of effector cells and a higher frequency of CM cells among both CD4+ and CD8+ T cells were found in PD patients than in HD patients. Thus, T cells were prone to a differentiated type in HD patients compared with PD patients. Besides, we found that HD patients had higher levels of inflammatory markers (such as IL-6 and TNF-a) and more serious azotemia than PD patients, so it is plausible that the difference of T cell phenotypes may be influenced by the altered microenvironment in HD and PD patients. Although it has been suggested that the improvement of inflammation status in ESRD patients by kidney transplantation could not change T cell subsets [22], the patients in that study included induction therapy via immunosuppression, which contributed to the exhaustion of T cells with replicative senescence [16].

Naïve T cells play key roles in maintaining the adaptive immunity function, and its loss would lead to a poor vaccine response and susceptibility to infection [23, 24]. Thus, the maintenance of naïve T cells may be pivotal in ESRD patients. With respect to naïve T cells in PD patients, the alteration was not consistent: the lower in CD4+ and higher in CD8+, compared with those in HD patients. It is reported that iron overload was associated with increased risks for death, infection and CVD [25, 26]. Ferritin level, an indicator of iron store and systemic inflammation, was also found to be negatively related to monocyte telomere length, but did not influence the T cell subsets frequency in HD patients [20]. Although the serum hemoglobin level in HD patients was higher than PD patients in our study, we did not find significant difference of serum ferritin level between HD and PD patients (Table 1), so the iron status may not be the cause of the alteration of naïve T cell.

It has been reported that the number of CD4+ naïve T cells was maintained for up to 60 years and then dramatically decreased in subsequent years with aging, owing to a reduction in homeostatic proliferation ability [27]. IL-7 may also prompt the expansion of CD4+ naïve T cells [28, 29], while patients with CKD 5 stage usually has significantly lower serum IL-7 than healthy controls [10]. In the present study, we found that only decreased nPCR was independently associated with reduced CD4+ naïve T cell subset in PD patients. Thus the diminished immune response, due to decreased proportion of CD4+ naïve T cells, may contribute to the risk of infection and mortality in PD patients. As to CD8+ naïve T cells, aging and dialysis therapy (either PD or HD) may influence the differences of CD8+ T cells in the previous studies [5, 7]. In agreement with these studies, we also found that age was an independent factor negatively influencing CD8+ naïve T cells in PD patients.

In summary, age and dialysis modality were factors altering T cell subsets. Compared with HD, PD reduced the progression from naïve T cells to effector T cells. In PD patients, the frequency of CD4+ naïve T cells may be affected by nutrition status, while the frequency of CD8+ naïve T cells may only associate with age.

## Conclusion

In summary, age and dialysis modality were factors altering T cell subsets. Compared with HD, PD reduced the progression from naïve T cells to effector T cells. In PD patients, the frequency of CD4+ naïve T cells may be affected by nutrition status, while the frequency of CD8+ naïve T cells may only associate with age.

## Data Availability

The datasets supporting the current study are available from the corresponding author on reasonable request.

## Abbreviations

ALT: Alanine aminotransferase
AST: Aspartate aminotransferase
BUN: Blood urine nitrogen
CAPD: Continuous ambulatory peritoneal dialysis
CKD: Chronic kidney disease
CM: Central memory
EM: Effector memory
EMRA: Effector memory RA
ESRD: End-stage renal disease
HD: Hemodialysis
hsCRP: high-sensitive C-reactive protein
iPTH: Parathyroid hormone
PD: Peritoneal dialysis
SCr: Serum creatinine
WBC: White blood cells
β2-MG: β2-microglobin
